# Images in Clinical Neurophysiology: a new subsection

**DOI:** 10.1590/0004-282X-ANP-2021-e006

**Published:** 2021-06-23

**Affiliations:** 1 Massachusetts General Hospital Department of Neurology Boston MA USA Massachusetts General Hospital, Department of Neurology, Boston, MA, USA.; 2 Optum Care Medical Group Huntington Beach CA USA Optum Care Medical Group, Huntington Beach, CA, USA.

The subspecialty of Clinical Neurophysiology involves the assessment of function of the central, peripheral, and autonomic nervous systems as well as skeletal muscle using both clinical evaluation and electrophysiologic testing. The latter includes electroencephalography (EEG), magnetoencephalography (MEG), electromyography (EMG), nerve conduction studies (NCS), polysomnography (PSG), evoked potentials, and autonomic testing. Practicing in clinical neurophysiology requires a sound understanding of clinical neurology, normal neurophysiology, and the wide array of abnormal neurophysiologic findings that may be associated with different neurologic disorders.

It is often challenging for neurology trainees to acquire optimal knowledge in neurophysiology during residency given its high clinical demand coupled with the multitude of subspecialties within neurology. Similarly, trainees may not have the opportunity to be exposed to all facets of clinical neurophysiology thereby narrowing their experience as budding neurologists. In light of these system-based constraints, the *Arquivos de Neuro-Psiquiatria* recognizes the need for a new journal subsection exclusively focused on the field of clinical neurophysiology. We hope this subsection will expand trainees’ learning experience and ultimately improve patient care as well as potentially encourage some trainees to pursue further training in neurophysiology.

*Images in Clinical Neurophysiology* welcomes manuscripts with highly educational value related to the subspecialty of neurophysiology. Neurophysiologic content (educational images and/or videos of EEG, EMG, NCS, PSG, and evoked potentials) should be previously unpublished, interesting material highlighting clear examples of established observations curated for a trainee readership. Authors should also submit three multiple-choice questions along with answers related to the respective manuscript.

Authors may consider selecting cases of uncommon presentations of common neurophysiologic disorders or common presentations of uncommon neurophysiologic disorders. Additionally, describing artifacts that may be mistakenly interpreted as abnormal also has great educational value. Within the realm of EEG and epilepsy, authors may consider the following examples as reference:

- A case of typical notched delta pattern on EEG in a patient with Angelman syndrome^1^.
- A report of the “texting rhythm” on EEG associated with cortical processing related to the use of personal electronic devices^2^.
- A description of snoring-related artifact, which was previously thought to arise from the cortex overlying the amygdala (“limbic spindles”)^3^.

Within the realm of electrodiagnostic testing in neuromuscular medicine, the following examples may serve as excellent teaching tools in highlighting the technical aspects of testing as well as the importance of a keen clinical correlation:

- The presence of ‘pseudo-conduction block’ without other demyelinating features in vasculitic mononeuritis multiplex, where a conduction block is manifested by focal infarction and axonal loss rather than the typical demyelinating mechanism^4^.
- The lack of temporal dispersion and conduction block with a predominance of prolonged distal latencies in anti-MAG demyelinating neuropathy, which provides a distinguishing feature from CIDP (chronic inflammatory demyelinating polyradiculopathy)^5^.
- The evaluation of anatomical variants, such an accessory deep peroneal nerve, in case of peroneal neuropathy resulting in foot drop with preservation of toe extension^6^.

We hope to attract outstanding and highly educational neurophysiology manuscripts ideally led by trainees from across the globe. We are truly grateful to the *Arquivos de Neuro-Psiquiatria* for creating this new subsection in the journal. We hope *Images in Clinical Neurophysiology* will become a unique educational resource and serve to (i) supplement neurophysiology education on an international level and (ii) inspire neurology trainees to embrace the fascinating field of neurophysiology.

## References

[1] 1. Nascimento FA, Thiele EA, Thibert RL. Teaching neuroimages: notched delta and angelman Syndrome. Neurology. 2021 May; 10.1212/WNL.0000000000012201. https://doi.org/10.1212/WNL.0000000000012201 10.1212/WNL.0000000000012201 34031203

[2] 2. Hanrahan B, Tatum 4^th^ WO. Teaching NeuroImages: Texting rhythm: A common EEG finding in the era of smartphone use. Neurology. 2020 Dec;95(24):e3454-55. https://doi.org/10.1212/WNL.0000000000010757 10.1212/WNL.0000000000010757 32887773

[3] 3. Sheikh Z. Snoring-related artifact: scalp EEG correlate of historical “limbic spindles”. Epileptic Disord. 2021 Feb;23(1):201-2. https://doi.org/10.1684/epd.2021.1241 10.1684/epd.2021.1241 33602659

[4] 4. McCluskey L, Feinberg D, Cantor C, Bird S. “Pseudo-conduction block” in vasculitic neuropathy. Muscle Nerve. 1999 Oct;22(10):1361-6. https://doi.org/10.1002/(sici)1097-4598(199910)22:10<1361::aid-mus4>3.0.co;2-1 10.1002/(sici)1097-4598(199910)22:10<1361::aid-mus4>3.0.co;2-1 10487901

[5] 5. Gondim F, De Sousa EA, Latov N, Sander HW, Chin RL, Brannagan TH. Anti-MAG/SGPG associated neuropathy does not commonly cause distal nerve temporal dispersion. J Neurol Neurosurg Psychiatry. 2007 Aug;78(8):902-4. https://doi.org/10.1136/jnnp.2006.111930 10.1136/jnnp.2006.111930 PMC2117731 17353253

[6] 6. Kayal R, Katirji B. Atypical deep peroneal neuropathy in the setting of an accessory deep peroneal nerve. Muscle Nerve. 2009 Aug;40(2):313-5. https://doi.org/10.1002/mus.21324 10.1002/mus.21324 19609929

